# Critical care for COVID-19 during a humanitarian crisis—lessons learnt from Yemen

**DOI:** 10.1186/s13054-020-03281-y

**Published:** 2020-09-23

**Authors:** James S. Lee, Aurélie Godard

**Affiliations:** 1 Medical Department, Médecins Sans Frontières - Operational Centre Brussels, Brussels, Belgium; 2 Medical Department, Médecins Sans Frontières - Operational Centre Paris, Paris, France

**Keywords:** Intensive care, Critical care, Humanitarian settings, COVID-19

On day 1, while assessing the site to plan patient flow, a newly recruited doctor approached and asked, “where do we get PPE? how does the centre work?” The response given, “I don’t know yet, but we will figure this out together.” Patients had already arrived.

In May 2020, Médecins Sans Frontières/Doctors Without Borders (MSF) opened three COVID-19 treatment centres (CTC) in Sanaa and Aden, Yemen [1]. We report our experience from rapidly setting up CTCs with intensive care units (ICU). Working in humanitarian crises presents numerous contextual and cultural issues [2], but providing critical care in a war-torn country during a pandemic has further challenges. In the first week of opening the Aden CTC, a surge of war-wounded resulted in a mass-casualty plan activation at the MSF Aden trauma hospital, which functioned as our COVID-19 response support base. Additionally, some hospitals were closed from fear of COVID-19, resulting in the Aden CTC becoming rapidly overwhelmed and a second CTC opening. Resource constraints, high influx of patients, and societal pressures were encountered in all 3 CTCs, requiring that lessons learnt be applied in real-time.

MSF’s three CTCs included wards and ICUs. Invasive mechanical ventilation (IMV) received global attention but is only the visible “tip of the iceberg” for COVID-19 care. A full package of critical care includes, but not limited to, critical care trained staff, allied health and logistics staff, clinical mentoring for juniors, biomedical equipment, oxygen, medications, and a reliable supply chain.

Our ICUs emulated a closed-unit model with local nurses and generalist doctors supervised by an international intensivist and nurse. Each ICU had context-specific resource constraints resulting in differences in the package of care related to equipment (ultrasound), investigations (laboratory, x-ray), oxygen supply, nutrition, medications, and staff (specialist doctors, nurses, physiotherapists, social workers, pharmacists, logisticians). Due to limited ICU beds, many critically ill patients remained in the ward where the maximal oxygen therapy was a non-rebreather mask (NRM) combined with a regular nasal cannula. This double oxygen set-up, in addition to prone positioning, successfully treated some patients, avoiding the need for ventilatory support.

All three CTCs had rapid increases in admissions and community/contextual pressure to open immediately leaving no time for pre-opening training. Local intensivists were not available and recruiting internationally was difficult, as the pandemic has increased the need for intensivists/ICU nurses worldwide, many who are obliged to work in their home country. Closure of airports/borders and security constraints limited the ability to move staff and supplies. This placed further pressure on local staff, many of whom had no or limited ICU experience. Routine ICU care, such as ventilator settings, ventilator-associated pneumonia prevention bundles, infusion pump usage, and early mobility, amongst others, were unfamiliar. Clinical protocols were developed and taught on-the-job. Prone positioning had never been performed locally, but successfully taught in all 3 ICUs. Teaching critical care concepts within a few days (which typically take years of training) was challenging enough, but further complexity was added by simultaneously managing patients with a new disease, where medical knowledge of COVID-19 was evolving daily.

The aim of MSF’s COVID-19 response in Yemen was to provide oxygen to the maximum number of patients possible, irrespective of ventilator capacity. Our CTCs were new structures without centralized oxygen, but solutions developed. In the first days at one ICU, each ventilator was attached to one oxygen cylinder, which meant the patient was deprived of oxygen when the cylinder was changed. Malfunctioning regulators delivered too much pressure, which damaged the ventilator and/or did not accurately measure the amount of oxygen remaining; thus, staff did not know when the cylinder was empty until the ventilator detected a sudden drop in FiO2. To overcome the above problems, a Y-connection circuit with 2 cylinders was created, ensuring that a ventilated patient was never deprived of oxygen.

Providing critical care requires a steady supply of medications, but supply challenges were numerous. We were unable to rely on MSF’s usual mechanisms for international procurement. A shortage of analgesia/sedation/neuromuscular blocking agents in addition to inconsistent oxygen supply forced us to adapt. When the medications for IMV were unavailable, non-invasive ventilation (NIV) was favoured—and sometimes the only option (high flow nasal cannula oxygen was unavailable). Bilevel positive airway pressure was commonly used because many patients presented late with silent hypoxia and increased work of breathing, requiring inspiratory pressure support. Initially, we did not have NIV masks and used Ambu-bag masks with a bandage wrap to secure them. This apparatus frequently had a poor mask seal, resulting in excessive oxygen use further straining our supply issues, but was life-saving in some cases. Our protocol for NIV in COVID-19 incorporated a weaning schedule alternating NIV with NRM by gradually decreasing-increasing the time on NIV-NRM over days. This avoided intubation in some patients who survived to ICU discharge after a mean 5.3 days of NIV (range 2 to 9 days). Overtime, IMV medications became available locally and sometimes the quality was uncertain (e.g., ineffective atracurium likely due to a lack of cold chain storage), but there was no alternative option. A consistent supply was needed and contributed to successful application of IMV. Survivors of IMV required a mean 19.3 ventilator days (range 8 to 33 days).

Patient outcomes for the first 6 weeks of operations for one ICU are shown in Fig. 1, but the high crude mortality provides an incomplete picture of outcomes, as quality of care improved overtime. Additionally, there were important secondary benefits from introducing critical care with IMV. Patients inside and outside of the ICU benefited overtime due to an increase in knowledge and awareness from monitoring, increase in staff skills, and ability to provide a higher level of care. Multidisciplinary teamwork was strongly encouraged in the ICU and this effect extended to the ward, where the ICU team frequently assisted in patient care.

**Fig. 1:**
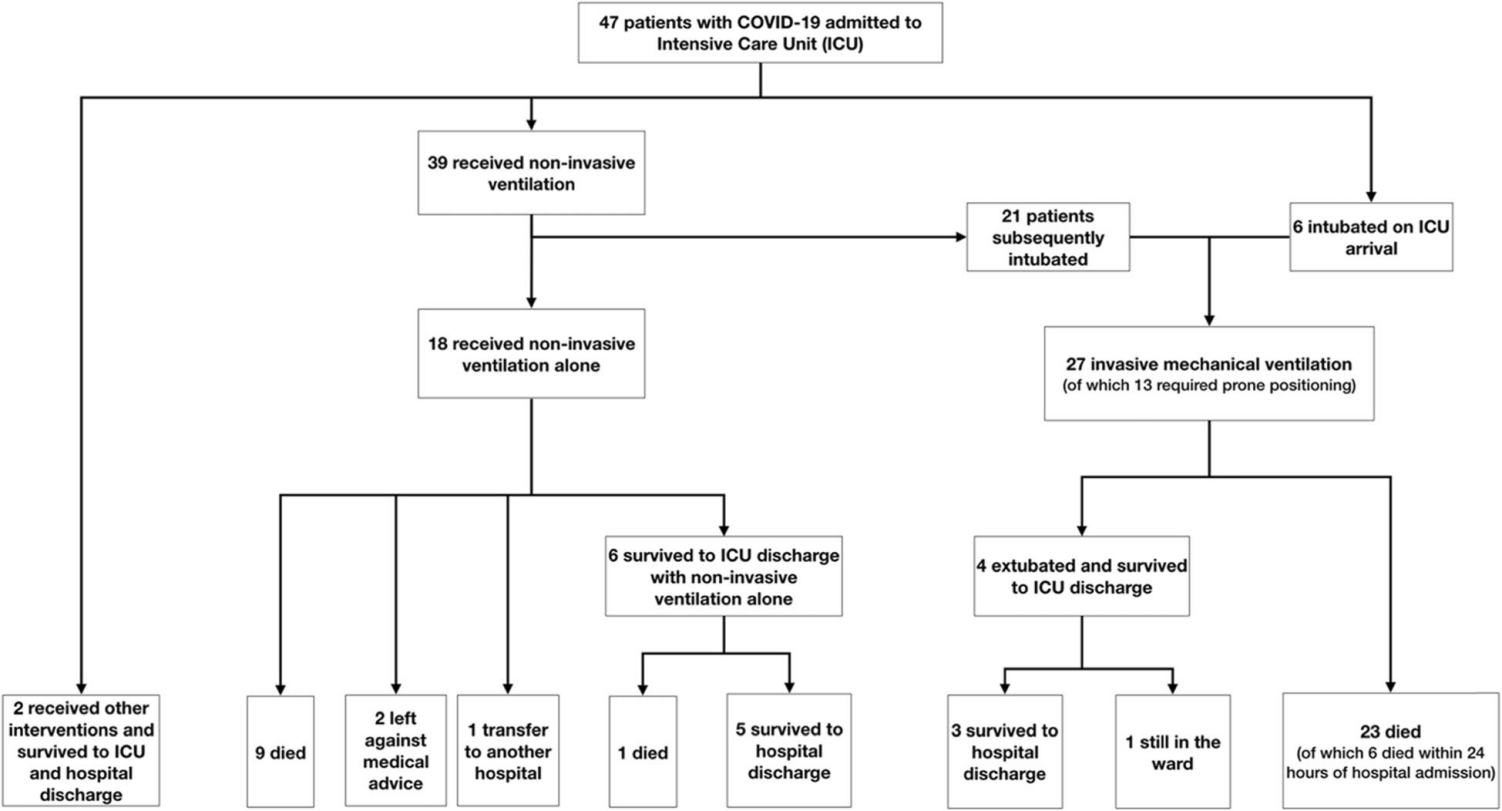
Confirmed COVID-19 (by PCR or CT scan) admitted to one intensive care unit (ICU) from 1 June 2020 to 12 July 2020

In summary, providing critical care with IMV for patients with COVID-19 during a humanitarian crisis in a war-torn country such as Yemen is feasible but requires implementation of a full package of care adapted to the context.

## Data Availability

Routine monitoring data was assessed as part of routine field operations. Data sharing is not applicable to this article as no datasets were generated or analysed for this article.

## Abbreviations

CTC: COVID-19 treatment centre
ICU: Intensive care unit
IMV: Invasive mechanical ventilation
MSF: Médecins Sans Frontières/Doctors Without Borders
NIV: Non-invasive ventilation
NRM: Non-rebreather mask
